# Primary Synovial Sarcoma of Kidney: A Rare Differential Diagnosis of Renomegaly

**DOI:** 10.1155/2014/657497

**Published:** 2014-12-24

**Authors:** Gaurang Modi, Irappa Madabhavi, Harsha Panchal, Asha Anand, Apurva Patel, Sonia Parikh, Swaroop Revannasiddaiah

**Affiliations:** ^1^Department of Medical and Pediatric Oncology, GCRI, Ahmedabad, Gujarat 830016, India; ^2^Department of Radiotherapy, Government Medical College, Haldwani, Nainital, Uttarakhand 263129, India

## Abstract

Synovial sarcomas (SS) are classified as subgroup of soft tissue sarcomas affecting mainly extremities of young adults. Primary SS of kidney are very rare tumours with poor prognosis. Though they have characteristic histology and immunohistochemistry (IHC) due to rarity of incidence it is difficult to diagnose them. Sometimes chromosomal rearrangement studies are required to confirm the diagnosis. We are presenting a case of 41-year-old male who was referred to our cancer centre for evaluation of left renal mass. CT scan of abdomen revealed a large left renal mass encasing the aorta. Biopsy of renal mass revealed poorly differentiated sarcoma and IHC was positive for vimentin, CD99, and BCL2 and negative for AE1, epithelial membrane antigen, and leukocyte common antigen. The patient was clinically inoperable as renal mass was encasing the aorta. So he was subsequently offered palliative chemotherapy in form of ifosfamide and adriamycin. CT abdomen shows partial response after 3 cycles of chemotherapy according to RECIST criteria.

## 1. Introduction

Synovial sarcomas (SS) are a group of soft tissue sarcomas (STS) affecting mainly young adults. The most common site of occurrence is in extremities. The term “synovial” is misnomer as tumour morphology and immunohistochemistry (IHC) do not correlate with normal synovium of joint [1]. Due to their rarity of occurrence it is a challenging task to diagnose them. Not only characteristic histology and IHC but also sometimes chromosomal rearrangement studies are required to confirm them.

Synovial sarcomas primarily originating in the kidney are very rare. Fewer than 50 cases of primary renal synovial sarcoma are reported till date [2]. Both primary renal synovial sarcomas and embryonal sarcomas of kidney have the same terminology described in literature.

## 2. Case Report

Forty-one-year-old male patient presented with pain in left lumbar region and macroscopic hematuria for 1 month. His past and family history is unremarkable. He is chronic tobacco chewer for 10 years and nonalcoholic. The patient was referred to our cancer centre based on abdominal ultrasonography findings of left renal mass. On examination the patient has normal height, weight, and body mass index for his age. His vitals were normal and performance score by ECOG (eastern cooperative oncology group) was 1.

**Figure 1:**
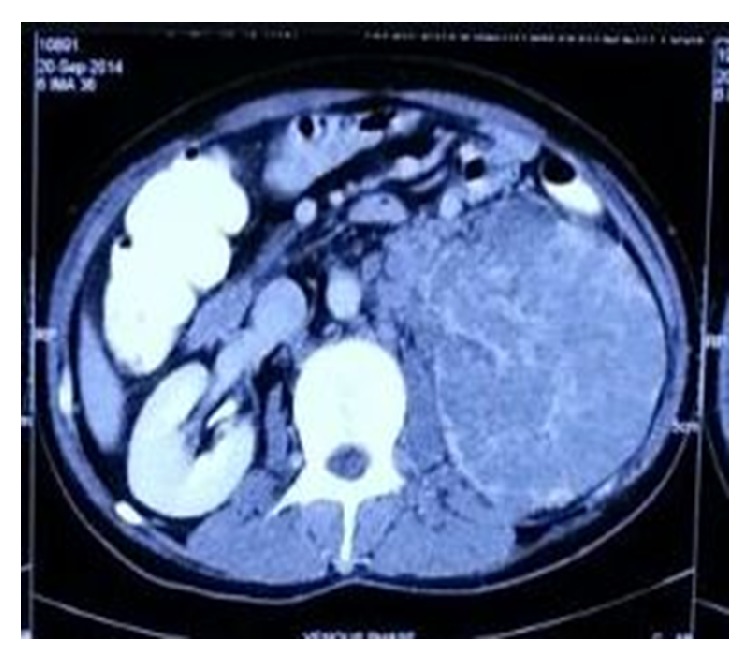
CT image shows enlarged left kidney and it is almost completely replaced with heterogeneously hypodense material.

Clinically nontender palpable mass was felt over left lumber fossa of around 5 × 5 cm with smooth surface and hard consistency. Pallor was present in the sclera and no lymphadenopathy or icterus was found. CT scan findings show enlarged left kidney and it is almost completely replaced with heterogeneously hypodense material. There is hypodense filling defect noted in left renal vein extending up to inferior vena cava suggestive of tumour thrombosis (Figure 1). Lab investigations were normal except haemoglobin of 6.7 gm%, serum creatinine of 2.1 mg/dL, and serum BUN of 25 mg/dL.

**Figure 2:**
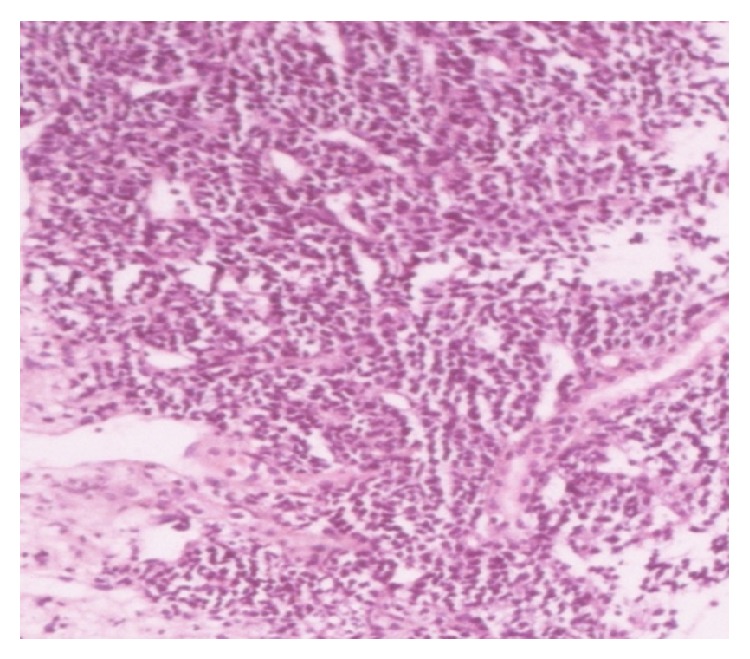
Low power view shows round to spindle cells with hemangiopericytoma pattern with areas of hyalinization in between.

**Figure 3:**
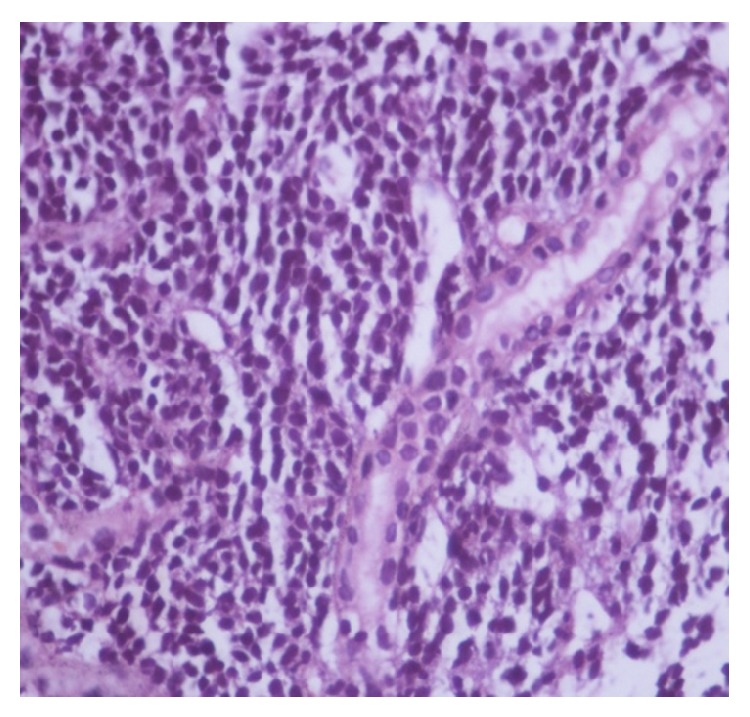
High power view shows entrapped normal renal tubules.

**Figure 4:**
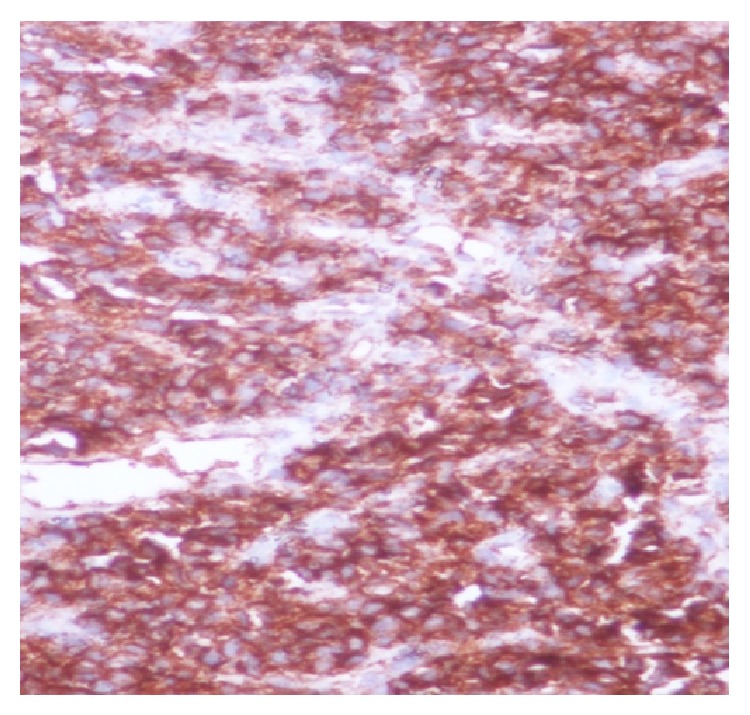
The figure shows CD99 positivity.

**Figure 5:**
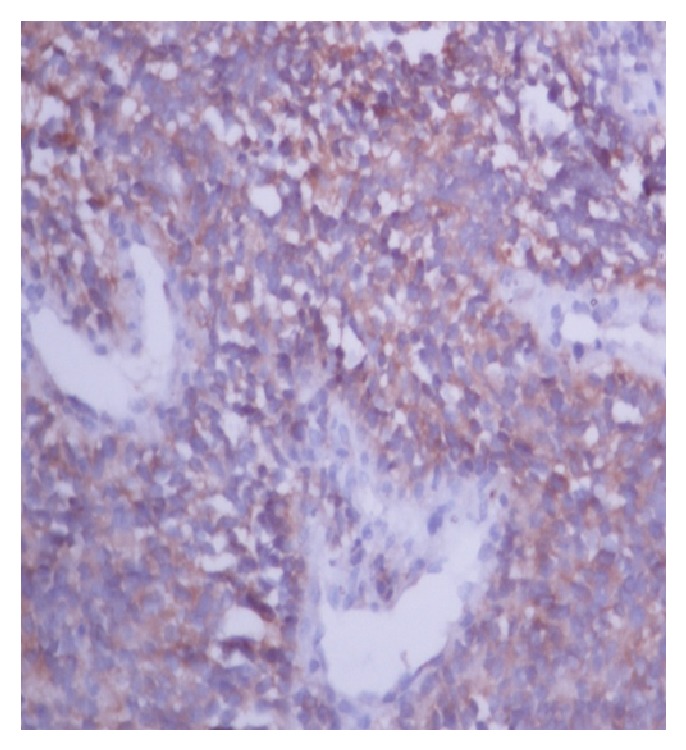
The figure shows BCL2 positivity.

Histopathological examination of the biopsy specimen from the left renal mass shows round to spindle cells with hemangiopericytoma pattern and area of hyalinization (Figure 2). High power view shows entrapped normal renal tubules (Figure 3). IHC was positive for CD99 (Figure 4), BCL2 (Figure 5), and vimentin and negative for AE1, epithelial membrane antigen (EMA), and leukocyte common antigen (LCA). According to morphological and IHC findings final diagnosis of primary renal synovial sarcoma was made.

The patient was clinically inoperable upfront according to urooncology surgeon. So he was subsequently offered palliative chemotherapy in form of ifosfamide and adriamycin. CT abdomen shows partial response after 3 cycles of chemotherapy according to RECIST criteria.

## 3. Discussion

Various STS primarily arising from kidney according to histology are leiomyosarcoma, liposarcoma, rhabdomyosarcoma, fibrosarcoma, and malignant fibrous histiocytoma [1, 3]. The most common three among them are leiomyosarcoma (40–60%), rhabdomyosarcoma, and malignant fibrous histiocytoma, respectively [4–7]. The incidence according to age ranges between 20 and 72 years with median age of 35 years. Rough male to female ratio is 1.7 : 1 [2]. Prevalence of primary renal SS is rare and comprises 1–3% of all malignant renal neoplasms [8]. The first case was reported in 1999 and Argani et al. published the first case of renal SS in 2000 [9].

Three histologic subtypes of synovial sarcoma are noted: monophasic, biphasic, and poorly differentiated. There is a clinical challenge to diagnose it. As spindle cell morphology its differential diagnosis would be sarcomatoid renal cell sarcoma, primary Ewing sarcoma of kidney, adult Wilms tumour, and undifferentiated carcinoma. The presence of both mesenchymal and epithelial markers is suggestive of synovial sarcoma at any site [9, 10]. When dilemma occurs even after IHC findings distinct chromosomal rearrangements study is needed [11]. Its unique chromosomal translocation is t(X; 18) (p11.2; q11.2) detected by fluorescent in situ hybridization (FISH) or reverse transcriptase polymerase chain reaction (RT-PCR) methods.

Primary renal SS have highly aggressive course and prognosis is poor. As tumor is very rare, no definite standard treatment guidelines are available at present. Treatment is usually based on the occurrence of scattered cases published in the world literature. Management of the patient by data is extrapolated from treatment of STS. Frontline surgery is advisable and there is no role of chemotherapy as curative intent. Chemotherapy can be given as palliative intent when upfront surgery is not possible for inoperable lesions or medically unfit patients or recurrence of the disease after upfront surgery. Still there are scattered case reports of adjuvant chemotherapy that is used in soft tissue sarcoma like doxorubicin, ifosfamide, and etoposide [2].

In our case the patient was inoperable so we started combination palliative chemotherapy in form of adriamycin and ifosfamide.

## 4. Conclusion

Primary renal SS are very rare type of STS with aggressive behaviour and poor prognosis. Surgery is the mainstay of treatment and adjuvant chemotherapy has some role. If not resectable role of chemotherapy is palliative.
